# Exploring regenerative coupling in phononic crystals for room temperature quantum optomechanics

**DOI:** 10.1038/s41598-024-63199-1

**Published:** 2024-05-29

**Authors:** Lukas M. Weituschat, Irene Castro, Irene Colomar, Christer Everly, Pablo A. Postigo, Daniel Ramos

**Affiliations:** 1 Optomechanics Lab, Instituto de Ciencia de Materiales de Madrid (ICMM), CSIC, 3, Sor Juana Inés de la Cruz, 28049 Madrid, Spain; 2 The Institute of Optics, University of Rochester, Rochester, NY 14627 USA

**Keywords:** Nanoscale devices, Optical materials and structures, Mechanical engineering

## Abstract

Quantum technologies play a pivotal role in driving transformative advancements across diverse fields, surpassing classical approaches and empowering us to address complex challenges more effectively; however, the need for ultra-low temperatures limits the use of these technologies to particular fields. This work comes to alleviate this problem. We present a way of phononic bandgap engineering using FEM by which the radiative mechanical energy dissipation of a nanomechanical oscillator can be significantly suppressed through coupling with a complementary oscillating mode of a defect of the surrounding phononic crystal (PnC). Applied to an optomechanically coupled nanobeam resonator in the megahertz regime, we find a mechanical quality factor improvement of up to four orders of magnitude compared to conventional PnC designs. As this method is based on geometrical optimization of the PnC and frequency matching of the resonator and defect mode, it is applicable to a wide range of resonator types and frequency ranges. Taking advantage of the, hereinafter referred to as, “regenerative coupling” in phononic crystals, the presented device is capable of reaching *f* × *Q* products exceeding 10E16 Hz with only two rows of PnC shield. Thus, stable quantum states with mechanical decoherence times up to 700 μs at room temperature can be obtained, offering new opportunities for the optimization of mechanical resonator performance and advancing the room temperature quantum field across diverse applications.

## Introduction

Quantum technologies have gained immense significance in recent years, revealing them as critical drivers of innovation^1^. These technologies leverage fundamental quantum phenomena like superposition and entanglement to surpass the capabilities of classical systems. In that sense, quantum computing^2^ offers exponentially faster processing power, with applications ranging from financial portfolio optimization to drug discovery by using qubits to process and store information; quantum cryptography^3^ ensures secure communication through techniques like quantum key distribution (QKD) and by utilizing quantum principles to detect any spying attempts; quantum sensing and metrology offer unparalleled precision in sensing and imaging, promoting advancements in fields like medical diagnostics and environmental monitoring^4^; and quantum materials and nanotechnology, which explores materials with unique quantum properties, enabling the development of applications like quantum dots and nanophotonics^5^. What makes quantum systems unique is coherence, which refers to the property where quantum states remain well-defined and maintain their phase relationships over time^6^. In this regard, temperature plays a crucial role, as higher temperatures introduce thermal fluctuations that disrupt quantum states, causing quantum information to degrade rapidly. Thus, to preserve coherence, it is essential to maintain low temperatures in quantum systems that minimize thermal energy and associated fluctuations, allowing for precise manipulation of quantum states. It is significant for quantum computing, where low temperatures reduce errors and enable longer quantum gate operations^7,8^. However, achieving extremely low temperatures may not always be practical, and efforts are currently focused on mitigating the effects of temperature and develop more robust quantum systems^9^. Therefore, although steps are underway to build room-temperature quantum systems, low temperatures remain a critical requirement for many quantum technologies.

On this point, cavity optomechanics has become a hot topic in recent years after entering the quantum regime and pushing the limits of signal processing, computing, communication^10–14^, and sensing^15–22^. In most cases, achieving the quantum ground state of the optomechanical resonator necessitates cooling it to nearly absolute zero temperature. However, as discussed earlier, this process demands complex developments like dilution cryostats. Therefore, there is a growing interest in devising quantum devices that can operate at or near room temperature, broadening the scope of applications and eliminating the need for elaborate cooling methods. The quantum ground state of a mechanical resonator refers to the lowest possible energy level that the system can attain, defined as an occupancy number of phonons equal to or smaller than one in a particular mode. One approach to reaching that occupancy number involves increasing the product of the mechanical mode frequency (*f*) and the quality factor (*Q*) of the resonator:

1 $$f\times Q > \frac{{k}_{B}{T}_{Room}}{h}=6.25\times {10}^{12}\, \text{Hz}$$

where *f* is the mechanical mode frequency, *Q* the mechanical quality factor, *k*_*B*_ the Boltzmann constant, *T*_*Room*_ the room temperature, and *h* the Planck’s constant. By doing so, the system’s thermal energy is decoupled from the surrounding thermal bath, resulting in a dominant contribution from quantum fluctuations^23^. Great efforts were made to achieve extremely high *Q*-values by combining techniques such as soft clamping, strain engineering and dissipation dilution^24–27^. The two main approaches in nanofabrication involve stress relief techniques and phononic shielding to decouple the device from its thermal environment^28^, with optimal results achieved through a periodic structure creating an acoustic bandgap that restricts phonon propagation at the resonator's operating frequency, reducing radiative mechanical energy losses and environmental effects. Therefore, the final value of the *f* × *Q* dramatically depends on the geometry, reaching 10^15^ Hz in one-dimensional strings^26^; 10^14^ Hz in hierarchical structures^29^, phononic defect mode^27^, and membrane-in-the-middle devices^30^; and 10^13^ Hz in trampoline resonators^31^, single crystal diamond nanobeams^32^ and optomechanical fishbone crystals^33^.

In this study, we introduce a new approach for achieving high *f × Q* values by employing parametric amplification of the mechanical mode in an optomechanical double nanobeam resonator through regenerative coupling with a phononic defect mode. The presented work demonstrates that this technique can enhance the mechanical quality factor of the resonator by up to four orders of magnitude when compared to a conventional phononic crystal design. Consequently, phonon decoherence time experiences a significant increase, transitioning from nanoseconds to several hundred microseconds, even at room temperature, leading to enhanced single photon cooperativity, ${C}_{0}= 1595$.

### Optomechanical coupling

**Figure 1:**
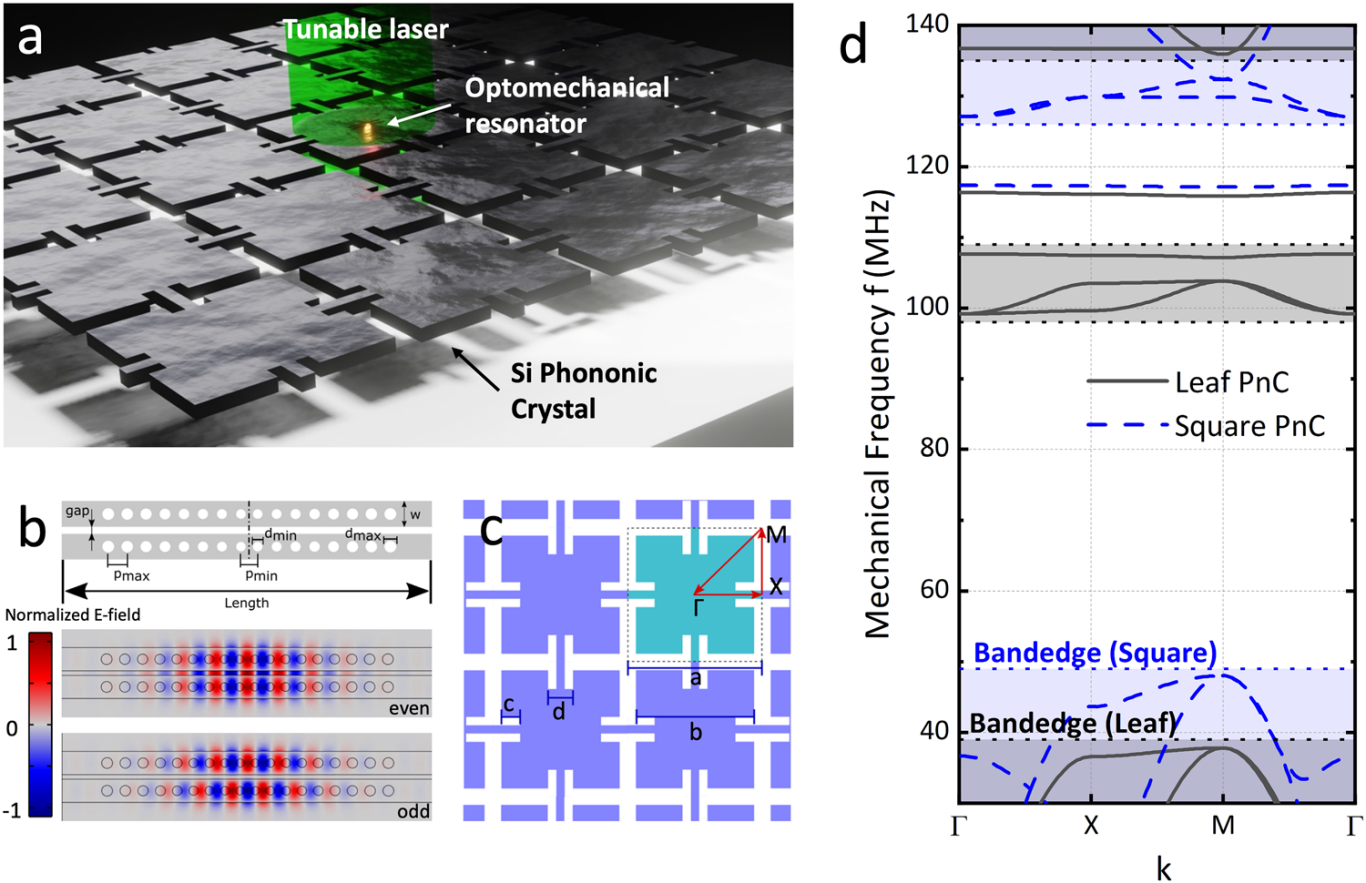
(**a**) Schematic depiction of the proposed quantum optomechanical device. The optomechanical resonator consisting of coupled photonic crystal nanobeam cavities (PCNCs) is embedded in a two-dimensional phononic crystal membrane. A tunable laser is used to probe the mode. (**b**) Schematics of the photonic crystal nanobeam cavities. FEM simulation of the normalized electric field distribution of the even (with a supermode shared between both beams) and odd optical modes of the proposed optomechanical resonator. (**c**) The unit cell of the Leaf design is shown in turquoise; red arrows depict the irreducible Brillouin zone (IBZ). Lattice constant a = 32.5 µm, b = 28.5 µm, notch depth c = 2.5 µm, notch width equals arm width d = 6 µm. (**d**) Simulated phononic band structure of the in-plane modes of the Leaf (black) and Square (blue) PnCs. The dotted lines and shaded areas highlight the bandgap edges and bands.

The proposed optomechanical resonator, illustrated in Fig. 1a, consists of coupled photonic crystal nanobeam cavities (PCNCs) embedded in a two-dimensional phononic crystal membrane. To proof the concept of the aforementioned regenerative coupling effect, the device was designed in silicon to take advantage of the mature fabrication technologies available. However, since silicon is known to exhibit high non-linear absorption^34^, the mechanical groundstate cannot realistically be achieved by optomechanical laser cooling alone. From a conceptual standpoint, the optical cavity can be visualized as a Fabry–Perot cavity at the wavelength scale, featuring photonic crystal mirrors that reflect and confine the nanobeam waveguide mode^35^. To avoid the impedance mismatch between the waveguide mode and the Bloch mode, the photonic crystal mirror is tapered by reducing the hole spacing, *s*, and radius to match the effective indices of the evanescent mirror Bloch mode *n*_*Bl*_ = *λ*/2*s* and the waveguide mode *n*_*wg*_ = 2.41. The cavities were designed using the Finite Element Method simulations (COMSOL) with 9 holes on either side of the cavity, tapering diameter and pitch linearly from *d*_*min*_ = 184 nm and *p*_*min*_ = 324 nm to *d*_*max*_ = 234 nm and *p*_*max*_ = 382 nm, in free-standing silicon nanobeams of thickness 220 nm and width 500 nm, Fig. 1b. The structure supports a fundamental TE-mode at *λ* = 1527 nm with an optical quality factor of *Q*_*opt*_ = 3.5 × 10^4^ and a small mode volume of about *V* = 0.02 λ^3^ and light can be coupled to it through vertical light injection. Apart from the mechanical coupling^36^, the two nanobeams can also interact through the optical force resulting from overlapping their optical resonances^37^. The optomechanical coupling constant, *g*_*om*_, quantifies the interaction strength between the optical field and mechanical motion within an optomechanical cavity^38^, reflecting how the resonance frequency of the cavity changes with the mechanical displacement, ${g}_{om}\equiv {\left.d{\omega }_{O}/dx\right|}_{x={x}_{0}}$. It serves as a figure of merit for comparing different optomechanical systems, offering insights into their interaction efficiency and the potential for precise control and measurement of mechanical and optical states. When comparing systems, *g*_*om*_ is evaluated not just by its magnitude but also in relation to the parameters of the specific system like decay rates and mechanical frequencies, allowing for a detailed assessment of the capabilities and effectiveness of each system in various applications, from quantum information processing to sensitive detection and actuation. It can be analytically calculated by perturbation theory^39^ as

2 $${g}_{om}=\frac{{\omega }_{0}}{4}\int dA\left(\overrightarrow{\psi }\cdot \widehat{n}\right)\left[\Delta \varepsilon {\left|{\overrightarrow{e}}_{\parallel }\right|}^{2}-\Delta \left({\varepsilon }^{-1}\right){\left|{\overrightarrow{d}}_{\perp }\right|}^{2}\right]$$

where $\overrightarrow{\psi }$ is the displacement field, equivalent to the shape of the different mechanical modes; $\overrightarrow{e}$ is the electric field; $\widehat{n}$ is the unit vector normal to the deflected surface of the PCNCs; $\Delta \varepsilon ={\varepsilon }_{1}-{\varepsilon }_{2}$, being *ε*_i_, *i* = 1,2 the dielectric constant of the structure and the surrounding medium, respectively, $\Delta \left({\varepsilon }^{-1}\right)={{\varepsilon }_{1}}^{-1}-{{\varepsilon }_{2}}^{-1}$; and $\overrightarrow{d}=\varepsilon \overrightarrow{e}$. Our system exhibits strong dispersion in the symmetric optical mode, with its wavelength dependent on the nanobeam separation. Conversely, the antisymmetric optical mode remains insensitive to the separation. Thus, the even optical mode proves highly sensitive to the collective motion of the nanobeams, so the slightest Brownian motion of the PCNCs significantly affects this mode^40,41^. We used FEM calculations to evaluate the optomechanical coupling constant between the even optical mode and the antisymmetric mechanical mode, considering the actual mode profiles. The *g*_*om*_ estimated through this perturbation theory calculation shows that the value for the antisymmetric mechanical mode is about 56.84 GHz/nm, for a gap size of 100 nm between the two nanobeams; see Supporting Information for further details.

### Phononic crystal simulation

**Figure 2:**
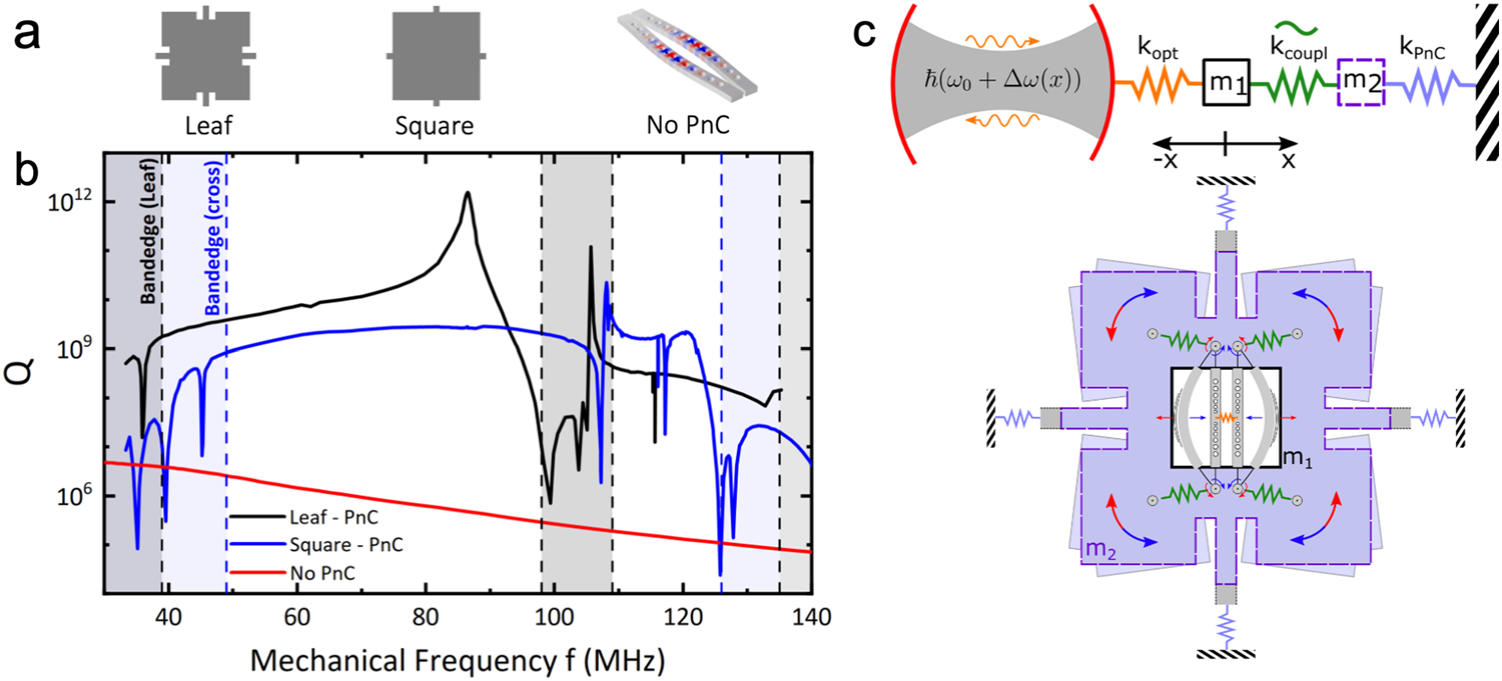
(**a**) Schematics of the different configurations of the PnC unit cell: The Leaf-PnC, the Square-PnC, and the double nanobeam resonator without any phononic shield. (**b**) Calculated mechanical Q-Spectrum of the optomechanical cavity's antisymmetric mechanical mode located within a phononic shield cell. Black: Leaf-PnC (notch depth = 2.5 µm), Blue: Square-PnC (notch depth = 0 µm), Red: optomechanical cavity without PnC. (**c**) Upper chart. Spring scheme illustrating the coupling between the nanobeams m_1_ (optical spring k_opt_, orange) and the phononic defect mode m_2_ (parametrically modulated coupling k_coupl_, green) and the coupling between the phononic defect mode and the phononic shield (k_PnC_, purple). Lower chart. sketch of the optomechanical double beam within the central cell of the PnC. The springs model the coupling of the antisymmetric double beam mode to the symmetric waving mode of the Leaf. For the sake of simplicity, the PCNCs are depicted vertically here. See the Supporting Information for a thorough analysis of the angular dependency of the nanobeams on the regenerative coupling feature.

The optomechanical cavity is then surrounded by a phononic crystal (PnC) of the same material with an acoustic bandgap centered at the frequency of the antisymmetric mechanical in-plane mode of the double nanobeams to isolate and decouple the resonator from external noise. The mechanical frequency *f* can be tuned by changing the length of the resonator. The design of the PnC originates from the well-established cross-structure^28^. Our design resembles the shape of a so-called “crutch-cross”, where another straight bar is attached at the end of each limb, Fig. 1c, creating a fractal-like structure. From now on, these two designs are referred to as “Square” and “Leaf” due to the similarity in their geometries when considering the material segments. The unit cell has the following geometrical parameters*: a* = 32.5 µm, *b* = 28.5 µm, *c* = 2.5 µm, *d* = 6 µm and a thickness of 220 nm. Due to the high unit cell lateral size/thickness ratio, the structure displays a quasi-bandgap, only confining mechanical in-plane modes^27^. The band dispersion diagram of the Leaf and Square designs for the same parameter *a* are displayed in Fig. 1d. For the calculation, we have used FEM simulations based on the Bloch theorem^42,43^, in which the governing field equations are given by

3 $$\sum_{j=1}^{3}\frac{\partial }{\partial {x}_{j}}\left(\sum_{l=1}^{3}\sum_{k=1}^{3}{c}_{ijkl}\frac{{\partial }^{2}{u}_{i}}{\partial {t}^{2}}\right)=\rho \frac{{\partial }^{2}{u}_{i}}{\partial {t}^{2}}, i=\text{1,2},3$$

where *ρ* is the mass density, *u*_*i*_ is the displacement, *t* is the time, *c*_*ijkl*_ are the elastic constants, and* x*_*j*_ (*j* = 1, 2, 3) represents the coordinates *x*, *y*, and *z*, respectively. By considering the system periodic along the x and y directions, according to the Bloch theorem, the displacement field can be expressed as $u\left(r\right)={e}^{i\left(kr\right)}{u}_{k}\left(r\right)$, where *k* = (*k*_*x*_*, k*_*y*_) is the wave vector limited to the first Brillouin zone and *u*_*k*_(*r*) is a periodic vector function with the same periodicity as the crystal lattice. The phonon dispersion and transmission eigenvalue equations can be written as $\left({\mathbb{K}}-{\upomega }^{2}{\mathbb{M}}\right)U=0$, where *U* is the displacement at the nodes and ${\mathbb{K}}$ and ${\mathbb{M}}$ are the stiffness and mass matrices, respectively. The Bloch theorem should be applied to the boundaries of the unit cell, yielding:

4 $$U\left(r+v\right)={e}^{i\left(k\bullet v\right)}U\left(r\right)$$

where *r* is located at the boundary nodes, and *v* is the vector that generates the point lattice associated with the phononic crystals. Single-crystal silicon is assumed as structural material with mass density ${\rho }_{Si}=2329\frac{kg}{{m}^{3}}$, Young’s modulus $E=170 \text{GPa}$ and Poisson’s ratio $\upnu =0.28$. The Square-design shows a quasi-bandgap from 49 to 125 MHz, with an isolated flat band ≈ 117 MHz. In comparison, the Leaf-design shows two bandgaps from 39 to 98 MHz and 109 MHz to 135 MHz with the flat band frequency slightly shifted to ≈ 116 MHz with a bandwidth of *∆f* ≈ 0.53 MHz. By introducing notches into the structure, the connecting bridges between neighboring cells are effectively lengthened, reducing the vibrational mode frequencies due to the extended leverage distance. The additional geometric degree of freedom enables the separate manipulation of the mechanical bands. By these means, several bands are pulled to lower frequencies, and the bandgap is divided into two. Because of the comparatively large unit cell, the double nanobeam resonator can be placed in a single cell of the PnC with variable lengths (ranging from 5 µm up to 12 µm, diagonal orientation) to span the frequency region of the phononic bandgap while oscillating at its fundamental in-plane mechanical mode. Since only the central cell of the PnC is modified, the symmetry is preserved, and the phononic shield stays intact. Additionally, by placing the optomechanical resonator within the cell, the mechanical mode frequencies of this particular cell are modified and pulled into the bandgap. Consequently, a phononic defect mode appears and oscillates at a reduced frequency compared to the initial mode frequency. Its mechanical energy dissipation is highly restricted due to the phononic shield effect imposed by the surrounding unmodified PnC cells^44^. We have analyzed how the PnC defect mode influences the asymmetric mechanical mode of the PCNCs, resulting in the emergence of regenerative coupling^45^. Employing finite element methods, we performed a parameter sweep of the length of the double nanobeam resonator to explore the frequency region of the bandgap and assess the quality factor (*Q*) of the antisymmetric in-plane mechanical mode within the phononic defect. To compute the mechanical quality factor an eigenfrequency analysis was conducted on the PCNCs surrounded by a single row of phononic shield supported by the substrate. The structure is contained in a hemispherical perfectly-matched-layer (PML) domain to capture the radiative mechanical losses into the substrate. Figure 2a shows the different configurations in which we have calculated the quality factor as a function of the optomechanical resonator frequency: The Leaf-PnC (black), the Square-PnC (blue), and the double nanobeam resonator without any phononic shield. The calculated *Q*-spectrum, the mechanical quality factor as a function of the frequency, is shown for all the different configurations in Fig. 2b.

## Results and discussion

### Regenerative coupling

As expected, it is evident that implementing any phononic shield enhances *Q* by several orders of magnitude when the resonator frequency falls within the bandgap. However, when the resonator frequency approaches a mode of the phononic crystal, a sharp drop in the quality factor is observed, indicating a coupling between the resonator and the phononic crystal. This coupling results in energy exchange and dissipation to the environment through the phononic shield. This effect is especially noticeable at the frequencies corresponding to the isolated flat bands, approximately around 117 MHz (as shown in Fig. 1d). Moreover, the *Q*-spectrum of the Leaf-PnC exhibits a distinct peak unrelated to any band, arising from the constructive interference between the coupled double beam motion and the phononic defect mode drawn into the bandgap, as previously described. The lower chart in Fig. 2c illustrates the coupling between the in-plane antisymmetric mechanical mode of the PCNCs and the phononic defect mode. The shifted defect mode originates from the flat band at approximately 107 MHz. This mode involves a symmetric angular motion of all four leaves around their "hinges" (regions with minimal displacement). Similarly, the nanobeams are hinged slightly inside the PnC cell at their anchor points. The material surrounding these hinges is a mechanical torsional spring, coupling the nanobeams and linking them to the phononic defect mode. The phononic defect mode undergoes a complementary motion to the optomechanical resonator by waving the "leaves" and the resulting mechanical stress induced by rotation near the hinges. This periodic modulation of the nanobeam coupling occurs at the same frequency and in-phase with the in-plane asymmetric coupled double nanobeam mode, facilitating energy exchange between the mechanical resonators (the defect mode and the PCNCs). In the upper chart of Fig. 2c, the antisymmetric nanobeam motion is optically excited using the optical spring^35,46^. Phonons that are optically excited through the antisymmetric mechanical mode of the beams resonate with the phononic defect mode, resulting in mutual amplification. Simultaneously, the phononic shield effectively impedes phonon dissipation to the substrate. When the mechanical eigenfrequency of the double beam coincides with the eigenfrequency of the phononic defect mode, their coupling reaches its maximum. As a consequence, a parametric amplification process occurs through regenerative coupling, wherein the optically pumped optomechanical resonator mode harmonically drives the phononic defect mode with minimal dissipation at its resonance frequency (as depicted in Fig. 2c). This process bears similarity to the technique of optical laser stabilization, wherein a relatively low-*Q* laser cavity (e.g., a diode laser) couples with an external passive high-*Q* reference cavity to enhance the optical quality factor of the laser output^47^.

**Figure 3:**
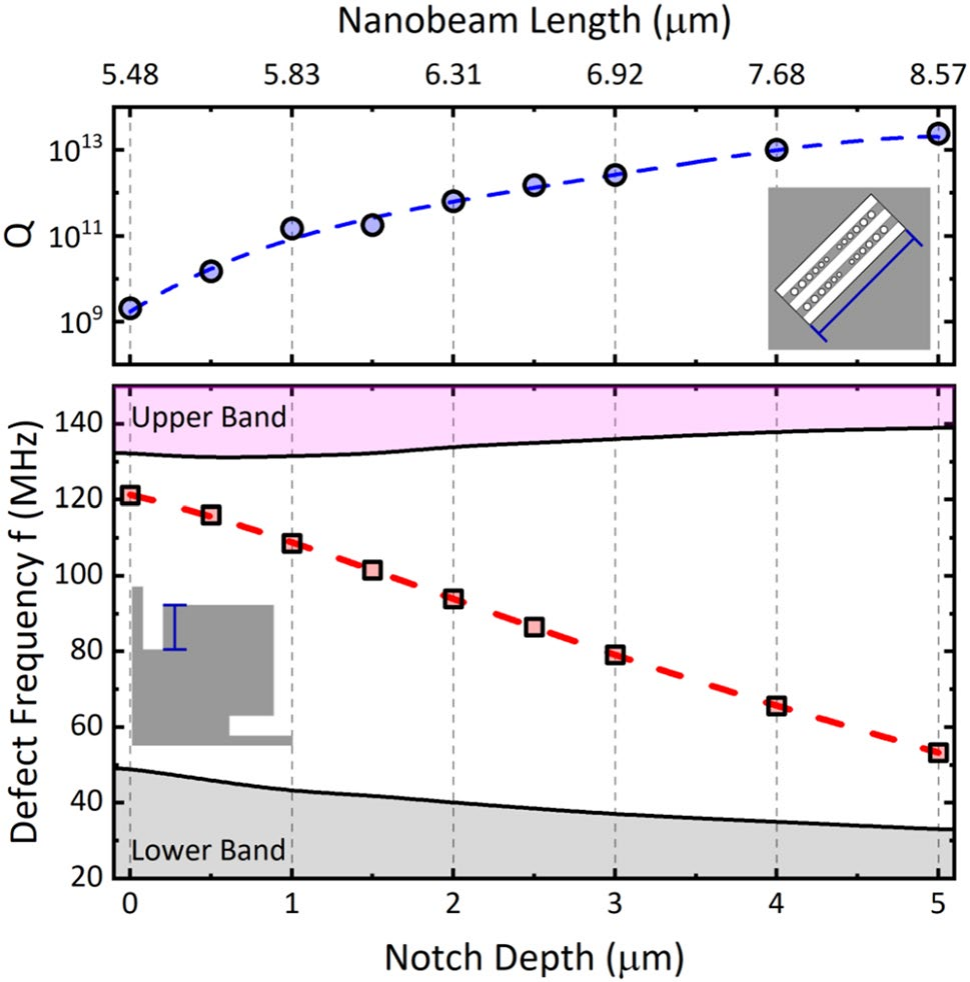
Evolution of the mechanical quality factor (upper chart) and Q-peak frequency (lower graph) within the phononic bandgap as a function of notch depth and nanobeam length. Note that the length of the nanobeam which maximizes the quality factor depends on the notch depth (see Supporting Information for further details).

To gain insights into this process, further simulations were conducted to investigate how modifying the unit cell geometry of the phononic crystal affects the mechanical bands and the quality factor. The lower chart in Fig. 3 displays the defect mode’s frequency shift (red symbols) (*Q*-peak) with increasing notch length. In contrast, the upper diagram (blue symbols) depicts its corresponding value, which increases by more than a thousand-fold. The *Q*-peak frequency linearly decreases as the notch depth grows, and its amplitude appears to saturate eventually. By comparing the 0 µm and 5 µm notch geometries and adjusting the mechanical resonator's beam length to match the *Q*-peak frequency, a remarkable four-order magnitude enhancement compared to the conventional square design can be achieved by exploiting the regenerative parametric amplification process between the phononic defect and the coupled optomechanical resonator. To attain even higher quality factors using this system, deeper notches, and longer nanobeams are required to accommodate the decreasing *Q*-peak frequency. However, these geometric parameters are limited by the physical size of the unit cell. An alternative approach to increase the mechanical quality factor is to add more rows of phononic crystal cells surrounding the modified central cell.

### Increased phononic shielding

**Figure 4:**
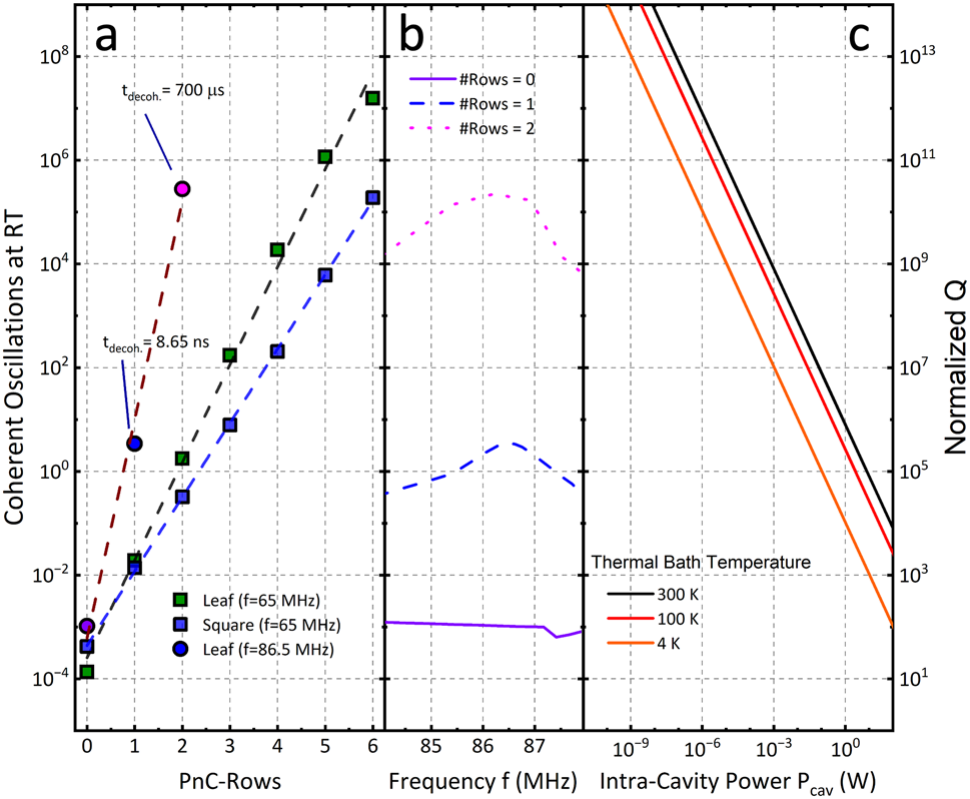
(**a**) Mechanical quality factor of the coupled nanobeam optomechanical resonator normalized on the mechanical quality factor of beams without phononic shield structure versus the number of PnC-rows surrounding the central defect. Black: Leaf-PnC (notch = 2.5 um) at 65.11 MHz, Red: Square-PnC at 65.11 MHz, Orange: Leaf-PnC (notch = 2.5 µm) at 86.4 MHz (Q-peak frequency). (**b**) Detail of the Q-peak spectra for the Leaf-PnC structure for increasing PnC rows. (**c**) Intra cavity optical power needed to reach the minimum achievable phonon occupation number as a function of the normalized Q for three different thermal bath temperatures: 300 K, black solid line; 100 K, red solid line; and 4 K, orange solid line.

This effect of extending the phononic crystal lattice was studied and the results are presented in Fig. 4. On the left side, Fig. 4a, the number of coherent oscillations and the normalized quality factor of the coupled optomechanical photonic crystal nanobeam cavities (PCNCs) are shown. Figure 4b shows the corresponding the *Q*-peak spectrum as a function of the number of rows in the phononic crystal. When the number of coherent oscillations equals one, the resonator undergoes exactly one oscillation before another phonon can enter the system from the thermal bath. This is the prerequisite to resolve a quantum state and is equivalent to *f* × *Q* = 10^12^ for room temperature (Eq. 1). It is calculated as follows

5 $${N}_{coh.Osc}=\frac{Qf\text{h}}{{k}_{B}T}.$$

The optomechanical damping rate ${\Gamma }_{opt}$ of the system is given by^48^

6 $${\Gamma }_{opt}=4{\left(\frac{{x}_{ZPF}}{{L}_{OM}}\right)}^{2}\frac{{{\upomega }_{R}}^{2}{\overline{n} }_{phot}}{\kappa }\frac{1}{1+{\left(\frac{\kappa }{4{\omega }_{m}}\right)}^{2}},$$

with ${n}_{phot}=4P/{\kappa \hslash }{\upomega }_{\text{opt}}$, x_ZPF_ the zero-point fluctuation amplitude, L_OM_ the optomechanical coupling length and κ the optical losses, see Supplementary Information for further details. By calculating the optomechanical damping rate, the mean phonon occupation number can be calculated as a function of the intra-cavity power using

7 $${n}_{mean}=\frac{{\Gamma }_{opt}{n}_{min}+{\Gamma }_{M}{n}_{th}}{{\Gamma }_{opt}+{\Gamma }_{M}}.$$

The Fig. 4c shows the necessary intra-cavity power (P_cav_) to cool the anti-symmetric mechanical mode of the PCNCs to the minimum achievable number of phonons for thermal bath temperatures of 300 K, 100 K and 4 K. With the better decoupling (higher mechanical Q) from the thermal bath, the phonon influx is decreased and less optical power is needed to achieve the minimum phonon occupation.

In the presented calculations, a nanobeam length of 7.7 µm within a unit cell featuring a notch of 2.5 µm is utilized to compare the performance of Leaf and Square-PnC, resulting in a mechanical resonance frequency of 65.11 MHz for the asymmetric in-plane mode. The simulated quality factor and the *N*_*coh.Osc*_ exhibit a linear increase in log scale with the number of rows in the phononic shield. The simulation suggests that leveraging the regenerative coupling mechanism (with *f*_*Res*_ = 86.5 MHz for notch depth 2.5 µm) can lead to a high-quality factor, achieving several coherent oscillations at room temperature with only a single row of the phononic crystal. The corresponding decoherence time ${t}_{decoh.}={N}_{coh.Osc}/2\uppi f$ (time the oscillator stays in its prepared quantum state) reaches 8.65 ns for one PnC row and exceeds 700 μs for two rows. Using the proposed system's values with two rows, we estimate a single-photon cooperativity of ${C}_{0}=1595$ and reach a quantum-cooperativity of ${C}_{q,300K}=2.1\times {10}^{-2}$ (see Supporting Information). However, it is essential to consider the model’s limitations, which assume no viscous damping with the surrounding medium and account only for clamping losses. As such, the obtained results may be overestimated and diminish when usual imperfections in the nanofabrication process, such as geometric variations, material inhomogeneity, and surface roughness, are considered. Nevertheless, incorporating more rows in the phononic crystal or using dynamic heterodyne control of the pumping laser may mitigate these potential effects.

### Robustness of the proposed design

Moreover, based on the *Q*-spectrum analysis (Fig. 2b), the *Q*-enhancement remains relatively robust against fabrication imperfections that may influence the resonator's frequency, owing to the broad width of the peak. For instance, a resonator with a length of *L* = 6.6 ± 0.02 µm (located at the *Q*-peak, with a notch length of 2.5 µm) exhibits a frequency error of ± 0.4 MHz while still attaining an enhanced *Q* value of approximately 10^12^. Similarly, with a notch length of 5 µm, a length error of ± 0.06 µm is sufficient to maintain the *Q* above 10^12^.

In addition to the *Q*-peak around ~ 86 MHz (as observed in Fig. 2b), another distinctive peak with a Fano-like shape appears at 105 MHz. Simulations indicate that this Fano-like peak emerges due to the diagonal orientation of the double beam resonator and its coupling with the diagonal stretching mode of the Phononic Crystal (PnC) (see Supporting Information). The unmodified unit cell mode, without the double beam resonator, is located at the flat band frequency of around 116 MHz. However, due to the cell modification by the nanobeams, the diagonal stretching mode of the original cell shifts to lower frequencies, similar to the phononic defect described earlier. Fano-shaped resonances are typically observed when two coupled harmonic oscillators are driven with a periodic force ^49^; in this case, one oscillator is the coupled optomechanical system, and the other is the PnC cell where it is positioned. By sweeping the coupled nanobeam resonance across the resonance of the PnC cell, a transition effect is revealed, where both resonances initially become out-of-phase, resulting in total suppression of their motion. Eventually, they end up in-phase after experiencing a *π*-phase jump, leading to the observed sharp peak. However, by altering the orientation of the double beam resonator from diagonal to vertical, thereby breaking the alignment with the diagonal stretching mode, the regenerative coupling is eliminated, reducing the *Q*-factor. More detailed results can be found in the Supporting Information. This secondary coupling feature demonstrates the applicability of the quality factor enhancement principle, exploiting regenerative coupling to a phononic defect mode to other modes through intelligent selection of resonator and PnC design.

## Discussion

In conclusion, our work demonstrates a technique to significantly enhance the quality factor of a mechanical resonator through coupling it with a confined defect mode of the surrounding phononic shield via frequency matching. By doing so, radiative mechanical energy losses are greatly reduced, enabling *f × Q* products surpassing the threshold for quantum measurements at room temperature $(f\times Q>\frac{{k}_{B}{T}_{Room}}{h}=6.25\times {10}^{12} \text{Hz})$ within the MHz frequency range. The application of the regenerative coupling technique to the presented optomechanically coupled nanobeam resonator shows that when comparing devices with a single phononic shield row an improvement by a factor of 10^4^ can be achieved over conventional phononic shield designs by simply tuning the PnC notch depth and resonator length. This is also the case even when the frequencies of the optomechanical cavity and the auxiliary cavity (phononic defect) are not precisely matched, which considerably relaxes the fabrication requirements. As a result, our Leaf-PnC shield using regenerative coupling shows promising potential for achieving higher mechanical quality factors with smaller footprint compared to the well-established Square-PnC (cross) design. Through additional PnC shield rows, stable quantum states with mechanical decoherence times in the microsecond range become feasible at room temperatures, enabling applications such as quantum memories^50^, quantum communication and signal processing^3^, single-photon and single-phonon sources^51^. Furthermore, coupling a mechanical resonator with a phononic defect mode to exploit the regenerative coupling effect can be extended to other resonator types and PnC structures through a specific selection of the involved resonators and mechanical modes, opening the door for new opportunities for advancing research and development of quantum optomechanics.

### Supplementary Information

Supplementary Information.

## Data Availability

All data generated or analysed during this study are included in this published article and its supplementary information files.
