# Supplementary material for: Exploring regenerative coupling in phononic crystals for room temperature quantum optomechanics
Source: Sci Rep. 2024 May 29;14:12330. doi: 10.1038/s41598-024-63199-1 (PMC11137142; doi:10.1038/s41598-024-63199-1)
Supplement: Supplementary file 1 — Supplementary Information. [file 41598_2024_63199_MOESM1_ESM.docx]

Supporting information

Exploring Regenerative Coupling in Phononic Crystals for Room Temperature Quantum Optomechanics

Lukas M. Weituschat^1^, Irene Castro^1^, Irene Colomar^1^, Christer Everly^2^, Pablo A. Postigo^2^, and Daniel Ramos^1,a^

^1^Optomechanics Lab, Instituto de Ciencia de Materiales de Madrid (ICMM), CSIC, 3, Sor Juana Inés de la Cruz, 28049 Madrid, Spain

^2^The Institute of Optics, University of Rochester, Rochester, NY 14627, USA

^a^e-mail: [daniel.ramos@csic.es](mailto:daniel.ramos@csic.es)

# Bandstructure Evolution

In the left panel of Fig. A, the full evolution of the mechanical bands of the Leaf-PnC with the notch depth is plotted. The right panel displays the mode shapes of the mechanical modes occurring in this frequency range.


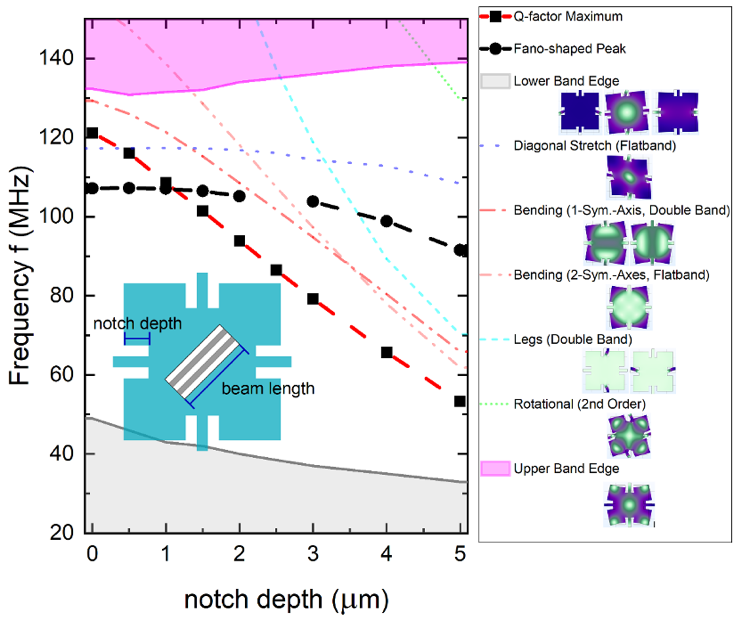


**Figure A.** Left panel: Evolution of the PnC-band structure with notch depth. Right panel: Mechanical displacement fields of the PnC modes.

The evolution of the mean band frequency of the mechanical modes matches well with the *Q*-spectra and bandstructures displayed in Fig.B (compare notch = 0, 2.5, 5 µm). The plot clearly shows the dependencies of the mean mode frequencies on the notch depth. This opens up the possibility to tailor the mechanical bandgap and behavior of the phononic crystal, e.g. through deepening of the notch, several modes are pulled from above the upper bandedge into the bandgap, effectively splitting it into two. Furthermore, with the separation of the waving mode (bending mode, biaxial symmetry, flatband) from the upper bandedge, the *Q*-factor enhancement of the double beam resonator emerges, its maximum shown as red dashed line. With deeper notch, the waving mode of the PnC approaches the *Q*-peak frequency, leading to a higher maximum and narrower peak width (Fig. B).


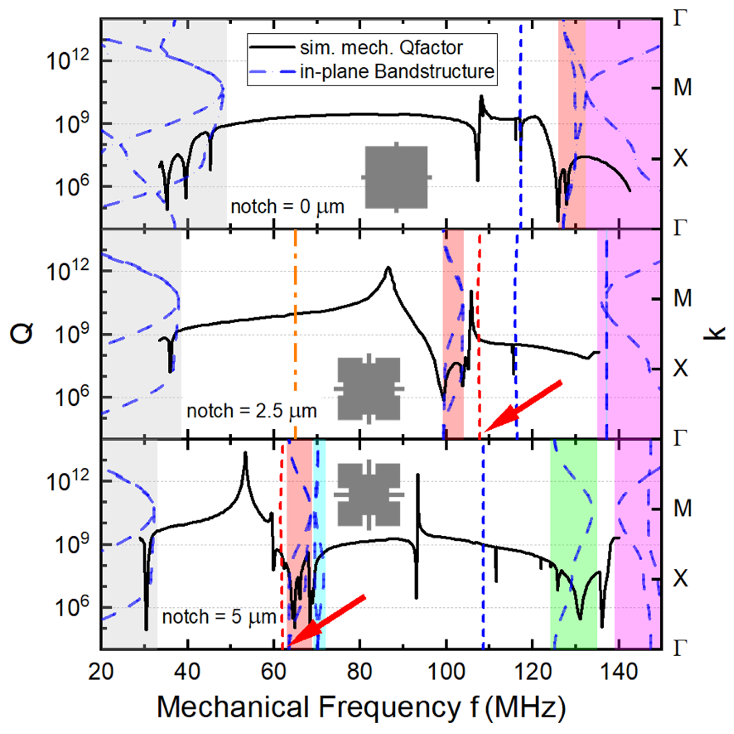


**Figure B.** Q-spectra and associated band structures of the Leaf-PnC with different notch depths. The biaxially symmetric bending mode (flatband) causing the Q-enhancement of the double beam resonator is marked in red dashed lines. Blue dashed lines mark the diagonal stretching mode (flatband), cause of the Fano-shaped peak in the Q-spectra.

This indicates an improvement of the coupling between the differential double beam motion and the PnC mode, displayed in Fig. C. Additionally, the frequency of the Fano-shaped peak (black dashed line) is plotted in Fig. A. Analyzing the displacement field of the double beam resonator at the frequency of the Fano-shaped peak reveals the coupling of the mechanical resonator with the diagonal stretching mode of the PnC. And, indeed, the evolution of Fano-shaped peak and diagonal stretching mode with the notch depth follow the same trend. The parallel behavior stems from the modification of the PnC-cell with the opto-mechanic resonator and its, therefore, reduced frequency compared to the original mode.


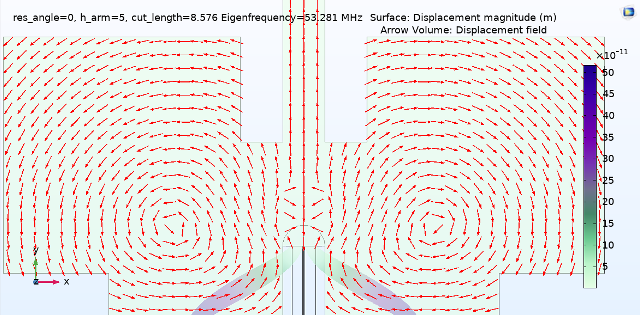


**Figure C.** Displacement field of the double beam resonator at the frequency of the Q-peak. Red arrows display the complementary waving motion of the leaves around their hinges, arising from the coupling with the differential mode of the PCNCs.

# Angular Dependence

Simulations of the double beam resonator’s *Q*-spectra with different angular orientations, help to uncover the origin of the Fano-shaped peak. Fig. E displays the *Q*-spectra of vertical and diagonal beams in a unit cell with a notch depth of 5 µm. A clear difference between the spectra can be seen at the frequency of the Fano-shaped peak. In the case of the diagonally aligned beams, the diagonal stretching mode of the PnC-cell is able to support the differential double beam mode, forming a combined mechanical supermode, which leads to the Fano-shape with a peak (dip) in the *Q*-spectrum for in-phase (out-of-phase) coupling. Fano-shaped resonances are typically observed when two harmonic oscillators are coupled and one of them is driven with a periodic force [1], in this case one being the double nanobeam and the other the PnC-cell where it is located. Sweeping the double beam resonance frequency (by adjusting the beam length) over the resonance of the PnC-cell, reveals a transition effect where both resonances are out-of-phase at first, resulting in total suppression of their motion, and then ending up in-phase with each other after experiencing a π-phase jump. By changing the orientation of the double beam resonator from diagonal to vertical, and, therefore, breaking the alignment with the diagonal stretching mode, the mutual coupling effect (regenerative coupling) is eliminated leading to a reduction of the *Q*-factor. Looking at the *Q*-peak due to the mutual coupling of the double beam with the waving mode of the PnC, the frequency stays unaffected while its shape changes with the orientation of the double beams. In fact, a second peak for vertical orientation of the beams becomes clearly distinguishable (≈ 59 MHz). Analyzing the displacement field of the modes near the dip between both peaks, reveals a phase jump between the waving motion of the Leaf and the differential motion of the beams. This leads to the realization that the dip between them indicates destructive interference between the two mechanical modes, resulting in the diminution of both. Because the coupling of the double beam to the waving of the leaves of the phononic defect mode is more distinct in the vertical orientation of the double beams, this destructive interference does not become so prominent for the diagonal arrangement.


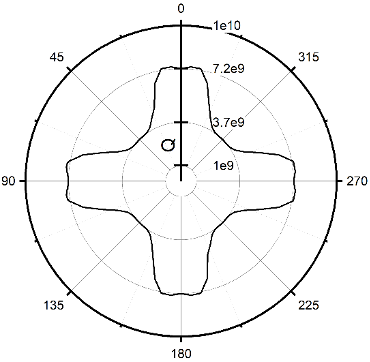


Figure D. *Q*-factor at different angular orientations of the PCNCs set within the PnC-cell.


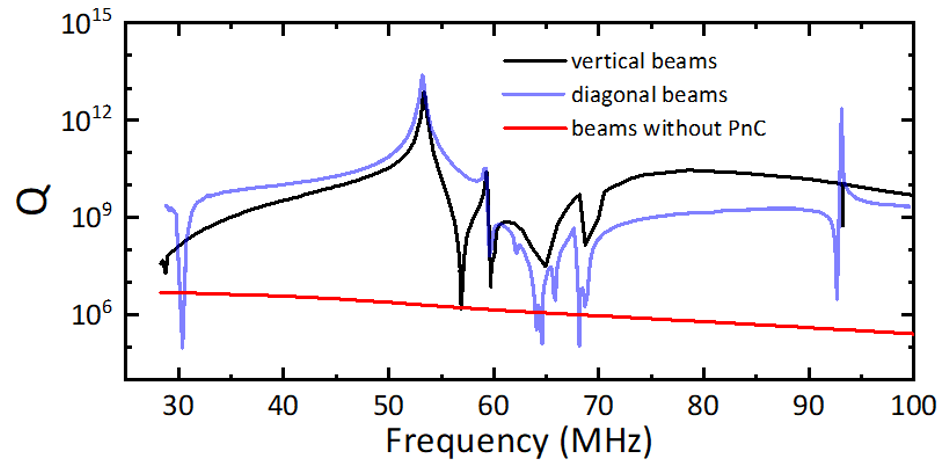


**Figure E**. Q-spectra of double beam resonator with vertical (black) and diagonal (blue) orientation within the Leaf-PnC (notch depth = 5 μm) and of beams without PnC (red).

# Minimum Phonon Number

Using optical simulations (COMSOL) of the mode shared between the photonic nanobeams, we estimate an $Q_{opt}=3.5*{10}^{4}$ at a resonance wavelength around $\lambda=1527 nm$. Therefore, our system operates in the optomechanical unresolved-sideband regime ($\kappa\gg\Omega_{m}$). Following Kippenberg et al.  [2], we can calculate the minimum phonon occupation achievable when employing laser-cooling techniques:

$$\begin{aligned} n_{min}= \frac{\kappa}{4\Omega_{m}}.\#\left( 1 \right) \end{aligned}$$

With

$$\kappa=\frac{\omega_{opt}}{Q_{opt}}= \frac{2\pi196.3277 THz}{3.5*{10}^{4}}=35.24 GHz$$

and the mechanical resonance mode at $f_{m}=85 MHz$

$\Omega_{m}=2\pi f_{m}=2\pi85 MHz=0.534 GHz$,

we estimate an achievable phonon occupation of $n_{min}=17$. To realize ground-state cooling, the opto-mechanical cavity needs to operate in the resolved-sideband regime ($\kappa\ll\Omega_{m}$), which demands an optical *Q*-factor of $Q_{opt}\geq5.78*{10}^{5}$.

# Opto-mechanical Coupling

The optomechanical coupling factor *g_OM_* describes the frequency shift per unit displacement:

$$\begin{aligned} g_{OM}=G=\frac{\delta\nu_{opt}}{\delta x}.\#\left( 2 \right) \end{aligned}$$

It can be estimated by calculating the effective opto-mech. coupling length L_OM_  [3]:

$$\begin{aligned} \frac{1}{L_{OM}}=\frac{1}{\nu_{opt}}\frac{\delta\nu_{\mathrm{opt}}}{\delta x}=\frac{1}{\nu_{opt}}g_{OM},\#\left( 3 \right) \end{aligned}$$

Which depends on the electric field $E$ and mechanical displacement field $Q$ as follows:

$$\begin{aligned} \frac{1}{L_{OM}}=\frac{1}{2}\frac{\int\mathrm{dA}\left( \frac{dQ}{d\alpha}\cdot\hat{n} \right)\left[ \Delta\varepsilon\left| E_{\parallel} \right|^{2}-\Delta\left( \varepsilon^{-1} \right)\left| D_{\perp} \right|^{2} \right]}{\int dV\varepsilon\left| E \right|^{2}}.\#\left( 4 \right) \end{aligned}$$

where the normalized displacement profile $\frac{dQ}{d\alpha}\cdot\hat{n}$, the electric field $E$ and electric displacement field $D$ distributions are obtained from eigenmode simulations using COMSOL. With simulated effective opto-mechanical coupling length $L_{OM}=3.45356 \mu m$ and resonance wavelength $\lambda=1527 \mathrm{nm}$ $\left( \nu_{opt}=\frac{c_{0}}{\lambda}=196.3277 \mathrm{THz} \right)$, we calculate an opto-mechanical coupling factor of

$$\begin{aligned} g_{OM}= \frac{\nu_{opt}}{L_{OM}}=\frac{196327.7 \mathrm{GHz}}{3453.56 \mathrm{nm}}=56.84\frac{\mathrm{GHz}}{\mathrm{nm}}. \#(5) \end{aligned}$$

# Effective Mass & Zero-Point Fluctuation

The effective mass of a mechanical resonator can be calculated from the mechanical mode volume $V_{m}$ and the density of the solid [3]:

$$\begin{aligned} m_{eff}=\rho V_{m}.\#\left( 6 \right) \end{aligned}$$

The mechanical mode volume $V_{m}$ depends on the mechanical displacement profile $Q(r)$, which is obtained from eigenmode simulation in COMSOL:

$$\begin{aligned} V_{m}=\int dV\left( \frac{\left| Q\left( r \right) \right|}{max\left( \left| Q\left( r \right) \right| \right)} \right)^{2}.\#\left( 7 \right) \end{aligned}$$

Simulation results yield for $V_{m}=0.5074 \mu m^{3}$, which leads to an effective modal mass of

$$m_{eff}=\rho_{Si}V_{m}=2329\frac{\mathrm{kg}}{m^{3}}\cdot5.074\cdot{10}^{-19}m^{3}=1.1817\cdot{10}^{-15}\mathrm{kg}.$$

From here, we can conclude a zero-point amplitude

$x_{ZPF}=\sqrt{\frac{\hbar}{2m_{eff}\Omega_{m}}}=\sqrt{\frac{\hbar}{2\cdot1.1817{\cdot10}^{-15}\mathrm{kg}\cdot2\pi\cdot86 \mathrm{MHz}}}=9.14\mathrm{fm}$ .

# Quantum Cooperativity

The single-photon cooperativity $C_{0}$ compares coupling strength $g_{0}$ to the optical and mechanical dissipation rates $\kappa and \Gamma_{m}$, respectively. It is a measure for the capability of the system to perform measurements at the Standard Quantum Limit (SQL) and is important for aspects such as the strength of optomechanically induced transparency [4]. A quantum-cooperativity $C_{q}=1$ gives the threshold to achieve phonon lasing and a cooperativity above unity is key requirement for efficient state transfer in hybrid quantum systems due to the interaction between photon and phonon being faster than mechanical thermal decoherence [4,5]. The single-photon cooperativity is calculated as follows:

$$\begin{aligned} C_{0}=\frac{4g_{0}^{2}}{\kappa\Gamma_{m}} ,\#\left( 8 \right) \end{aligned}$$

with $g_{0}=g_{OM}{\cdot x}_{ZPF}$ ($g_{0,Leaf}=519.5 \mathrm{kHz}$). Using $Q_{opt}=3.5*{10}^{4}$ and $Q_{m}={2.78*10}^{10}$ ($\kappa=\frac{2\pi196.3277 \mathrm{THz}}{3.5*{10}^{4}}=35.245 \mathrm{GHz}$, $\Gamma_{m}=\frac{2\pi85 \mathrm{MHz}}{2.78*{10}^{10}}=19.2 \mathrm{mHz}$ , respectively), a single-photon cooperativity of $C_{0}=1595$ is calculated. From this, the quantum cooperativity $C_{q}$can be estimated, using the formula:

$$\begin{aligned} C_{q}=C_{0}\frac{n_{opt}}{n_{th}},C_{q_{\max}}=C_{0}\frac{1}{n_{\mathrm{th}}},\#\left( 9 \right) \end{aligned}$$

where *n_opt_* is the number of photons in the cavity and $n_{th}=\frac{k_{B}T}{\hbar\Omega_{m}}$ the number of phonons in the surrounding thermal bath. The maximum (single) quantum cooperativity is reached for *n_opt_*=1. For the Leaf-structure, quantum cooperativities of $C_{q,300K}=2.1\cdot{10}^{-2},$ and $C_{q,4K}=1.62$ were calculated, scaling linearly with $Q_{opt}\mathrm{and} Q_{m}$.

# Mean steady-state phonon number

The mean steady-state phonon number $n_{mean}$ of the opto-mechanical system connected to a thermal bath with $n_{th}=\frac{k_{B}T}{\hbar\Omega_{m}}$ can be estimated as follows [6]:

$$\begin{aligned} n_{mean}=\frac{\Gamma_{opt}n_{min}+\Gamma_{M}n_{th}}{\Gamma_{opt}+\Gamma_{M}}, \end{aligned}(10)$$

with the mechanical damping rate $\Gamma_{M}$ and the opto-mechanical damping rate $\Gamma_{opt}$

$$\begin{aligned} \Gamma_{opt}=4\left( \frac{x_{ZPF}}{L_{OM}} \right)^{2}\frac{{\omega_{R}}^{2}\bar{n}_{phot}}{\kappa}\frac{1}{1+\left( \frac{\kappa}{4\omega_{m}} \right)^{2}}. \end{aligned}(11)$$

For the single-photon ($\bar{n}_{phot}=1$) opto-mechanical damping rate, we conclude a value of $\Gamma_{opt}=112 \mathrm{mHz}.$ Fig. F displays the mean steady-state phonon number versus temperature for different mechanical *Q*-factors.


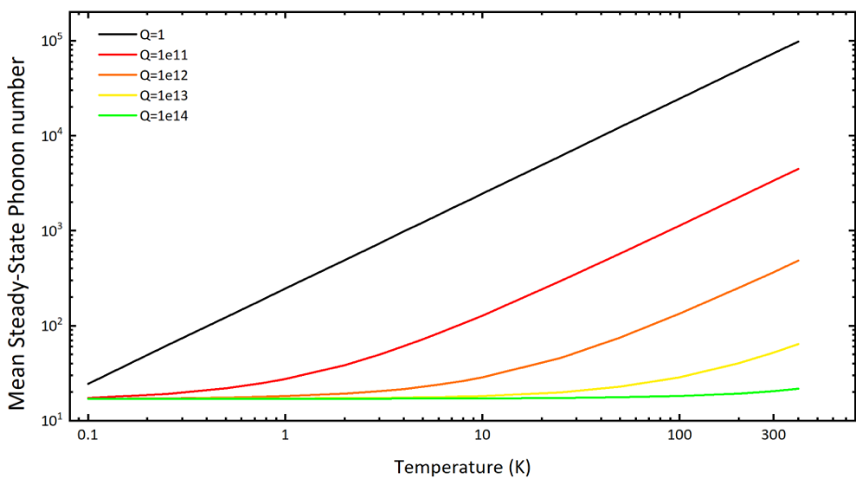


**Figure F.** Through laser-cooling achievable phonon number depending on the environmental temperature for mechanical *Q*-factors from 10^10^ to 10^14^, assuming an opto-mechanical coupling rate $\Gamma_{opt}=112 \mathrm{mHz}$ and minimum achievable phonon number $n_{min}=17$.

# References

[1] A. E. Miroshnichenko, S. Flach, and Y. S. Kivshar, *Fano Resonances in Nanoscale Structures*, Rev. Mod. Phys. **82**, 2257 (2010).

[2] kippenberg Vahala, *Cavity Optomechanics*, Opt. Express (2007).

[3] M. Eichenfield, J. Chan, R. M. Camacho, K. J. Vahala, and O. Painter, *Optomechanical Crystals*, Nature **462**, 78 (2009).

[4] M. Aspelmeyer, T. J. Kippenberg, and F. Marquardt, *Cavity Optomechanics*, Rev. Mod. Phys. **86**, 1391 (2014).

[5] A. H. Safavi-Naeini, D. Van Thourhout, R. Baets, and R. Van Laer, *Controlling Phonons and Photons at the Wavelength Scale: Integrated Photonics Meets Integrated Phononics: Publisher’s Note*, Optica **6**, 410 (2019).

[6] F. Marquardt, J. P. Chen, A. A. Clerk, and S. M. Girvin, *Quantum Theory of Cavity-Assisted Sideband Cooling of Mechanical Motion*, Phys. Rev. Lett. **99**, 093902 (2007).
